# Quantitative Proteomic Analysis and Evaluation of the Potential Prognostic Biomarkers in Cholangiocarcinoma

**DOI:** 10.7150/jca.29354

**Published:** 2019-07-05

**Authors:** Zengwei Tang, Yuan Yang, Jinduo Zhang, Wenkang Fu, Yanyan Lin, Gang Su, Yan Li, Wenbo Meng, Xun Li, Xiaodong Xie

**Affiliations:** 1 The First Clinical Medical School of Lanzhou University, Lanzhou 730000, China.; 2 Department of Special Minimally Invasive Surgery, The First Hospital of Lanzhou University, Lanzhou 730000, China.; 3 School of Basic Medical Sciences, Institute of Genetics, Lanzhou University, Lanzhou 730000, China.; 4 Cleveland Clinic, Department of Inflammation and Immunity, Cleveland OHIO 44195, USA.; 5 The second department of General Surgery, The First Hospital of Lanzhou University, Lanzhou 730000, China.

**Keywords:** cholangiocarcinoma, iTRAQ, PPP3CA, prognosis, bioinformarics

## Abstract

**Background & Aims:** Cholangiocarcinoma (CCA) patients have poor outcomes since the late diagnosis limits the benefits of surgery therapy and curative treatment options. The present study was designed to screen the biomarkers for CCA patients.

**Methods:** Quantitative iTRAQ proteomic analysis was used to identify differentially expressed proteins between CCA and pericarcineous tissue. We examined the expression profile of PRDX2, BGN, LUM, and PPP3CA in CCA tissue using immunohistochemistry. We further investigated the correlation between PPP3CA expression and the survival of CCA patients (n=91).

**Results:** 2,886 confidential proteins were identified by using the iTRAQ technique, 233 of which were differentially expressed. PRDX2, BGN, PPP3CA, and LUM were expressed in CCA tissue, whereas they were not expressed in choledocal cyst tissue except for LUM. PPP3CA was expressed in the cytoplasm of carcinoma cells in 22 cases (24.2%) of 91 CCA patients. Patients with PPP3CA-positive expression showed a significantly shorter survival period than did non-expressing patients (*P* = 0.030). The univariate analysis showed that tumor size (*P* = 0.002), vascular invasion (*P* = 0.001), histological grading (*P* = 0.011), and PPP3CA expression (*P* = 0.033) were statistically significant risk factors affecting the prognosis of CCA patients. The multivariate analysis demonstrated PPP3CA expression (*P* = 0.009) and vascular invasion (*P* = 0.012) were statistically significant independent risk factors of CCA patients.

**Conclusions:** The results suggested that the expression of PPP3CA in CCA patients is a new independent factor for poor prognosis and a useful prognostic predictor for patients with CCA.

## Introduction

Cholangiocarcinoma (CCA), a heterogeneity group of malignancies originating in the epithelia at various anatomic locations in the biliary tree, is the most common primary malignancy of the biliary tract 1. The median survival for patients with unresectable CCA is less than a year 2. The prognosis is considerably better for patients who have undergone radical resection of CCA, with five year-survival rates ranging from 20% to 40% 3, 4. The late appearance of its clinical presentation makes CCA difficult to detect at an early stage—even with combining non-specific biomarkers, as well as with using advanced imaging technology. Late diagnosis compromises the effectivity of therapeutic options, which are based on surgical resection and/or liver transplantation; whereas chemotherapies are virtually palliative, given the marked chemoresistance of this cancer 3, 5.

Carbohydrate antigen 19-9 (CA19-9) is widely used as a serological marker of CCA. However, the capability of CA19-9 as a diagnostic marker is limited due to the influence of co-existing inflammation in the biliary tract and due to the fact that carcinoma cells from Lewis-gene-negative cases do not produce CA19-9 6, 7. Furthermore, CA19-9 cannot detect the development and progression of CCA 3. Several specific CCA biomarker studies to overcome these limitations have been performed 8. Wisteria floribunda agglutinin sialylated-Mucin1 has been identified as a novel diagnostic indicator of CCA in bile with a high specificity in comparison with CA19-9 9, 10. In addition, Mucin1 also has been demonstrated as having prognostic value for CCA patients 11. However, these biomarkers have also been found to be expressed in other tumors such as breast cancer, pancreatic cancer, etc. 12. Therefore, it is considered very important to screen suitable prognostic and/or diagnostic biomarkers of CCA, and to discover potential treatment strategies for this intractable disease.

Isobaric tags for relative and absolute quantitation (iTRAQ) are characterized by high proteome coverage and labeling efficiency, and has no side effects on analytical or biochemical properties of the labeled proteins or peptides 13. Currently, iTRAQ-based quantitative proteomic analysis has been become a promising technology for identifying differentially expressed proteins. The aim of this study is to explore the differentially expressed proteins in paired CCA and pericarcinous tissue by means of iTRAQ technique in combination with liquid chromatography-mass spectrometry (LC-MS), focusing on identifying reliable biomarkers for the prediction of CCA patients' prognosis. Among the differentially expressed proteins identified using iTRAQ-based quantitative proteomic analysis, the expression profile of peroxiredoxin2 (PRDX2), biglycan (BGN), lumican (LUM), and serine/threonine-protein phosphatase 2B catalytic subunit alpha isoform (PPP3CA) in primary CCA tissue were examined using immunohistochemistry (IHC) assay. In addition, we found that PPP3CA expression in CCA patients is a very useful predictor for poor prognosis.

## Materials and Methods

### Selection of patients and collection of clinical samples

A total of six matched, fresh primary CCA and the pericarcinous tissues were collected and employed for iTRAQ followed by LC-MS. The clinicopathological features of these patients are summarized in** Table 1.** Of patients who underwent surgical therapy at The Department of Special Minimally Invasive Surgery, The First Hospital of Lanzhou University from April to June, 2017, all were postoperatively diagnosed as having had primary CCA. Gross appearance and histological type was confirmed by two pathologists in the Department of Pathology of The First Hospital of Lanzhou University.

Furthermore, paraffin-embedded tissues of 5 cases of choledochal cyst, as well as 91 paraffin-embedded CCA cases for immunohistochemistry (IHC), were obtained with informed consent from patients who underwent surgical therapy at The First Hospital of Lanzhou University from 2011 to 2016. The mean age of patients presenting with choledochal cyst was 9.2 years (range: 3—17 years), and all patients with choledochal cyst were confirmed by pathologists postoperatively. For cases of CCA patients, the corresponding clinicopathological parameters are summarized in **Table 2.** The experimental protocol was approved by the Ethics Committee for Human Research, Lanzhou University. The written consents were received from all participants in this study. The inclusion criteria of patients in this study were as follows: (1) pathologically diagnosed as primary cholangiocarcinoma; (2) without neoadjuvant or adjuvant radiotherapy; (3) underwent a surgical resection; (4) no distant metastasis at first visit; (5) with adequate clinical information and follow-up data; (6) no preoperative and/or postoperative life-threatening complications; and (7) clinical data, including tumor differentiation and T/N stage, were available for all patients. The pathological stage of each cancer at the time of operation was identified by using the 7th edition of Classification of American Joint Committee on Cancer 4.

### Protein extraction and purification

Tissue samples were sectioned and thawed on ice. The tissues were ground in liquid nitrogen and extracted with lysis buffer (containing 7 mol/L urea, 2 mol/L thiourea, 4% 3-(3-cholamidopropyl) dimethylammonium propane sulfonate (CHAPS), 65 mmol/L dithiothreitol, and 0.1 mmol/L phenylmethylsulfonyl fluoride) combined with 4% phenylmethanesulfonyl fluoride (PMSF). The samples were then sonicated (80W) on ice for 3min (0.8-second sonication, followed by an 0.8-second break). For protein precipitation, pre-chilled acetone was added to the samples (5ml acetone:1ml samples), and the mixture was stored overnight at -20℃. The resulting suspension was centrifuged for 10 min at 12,000 x g at 4℃. After removing the supernatant, 2 ml pre-chilled acetone was added to the precipitate, and the mixture was centrifuged twice for 15min at 12,000 x g at 4℃. The pellet was air dried at room temperature after removing the supernatant. Then the precipitate was dissolved in 0.5ml 1M triethylammonium bicarbonate buffer (TEAB; Sigma-Aldrich, Australia) and centrifuged for 15min at room temperature. The supernatant was then transferred to a fresh 1ml tube, and the protein concentration was quantified by using the Bradford Protein Assay (Tiangen, Beijing, China).

### iTRAQ labeling and peptides fractionation

The iTRAQ Labeling procedures were conducted according to the manufacturer's instructions (AB SCIEX, Shanghai, China). 100ug proteins of each group were precipitated with fivefold acetone at -20℃ for 1 h. Then, the mixture was centrifuged for 10min at 12,000 x g at 4℃. After removal of the suspension, the protein was dried using a vacuum centrifuge. Then, the protein was resuspended in 50μl dissolution buffer, reduced by 4μl reducing reagent for 1 h at 60℃, and then alkylated by 2μl cysteine blocking reagent for 10 min at room temperature. Protein samples were then digested with 50μl trypsin (50ng/μl) at 37℃ for 12 h. Tryptic peptides were dried by vacuum centrifugation and labeled with the iTRAQ regents at room temperature for 2 h. Afterward, 100μl distilled water was added to stop the reaction, the samples were mixed with equal amounts, and then the dried samples using a vacuum centrifuge were left for isolation and identification.

### Strong cation exchange (SCX) chromatography-based fractionation

SCX chromatography-based fractionation and LC-MS/MS were conducted by Sangon Biotech (Shanghai) Co., Ltd. SCX chromatography was performed as previously described 14. The peptides were fractionated on an apolysulfoethyl A column (2.1×150 mm 5μm, 300 Å, Poly LC, Columbia, MD, USA) by using an Agilent 1200 HPLC system (Agilent, Beijing, China). Firstly, the mixed peptides, which were dissolved in 110μl Nano-RPLC buffer A (0.1% formic acid and 2% ACN), were further separated by Agilent 1200 HPLC (Agilent, Beijing, China) on the secondary RP analytical column (Analytical Guard Column 4.6×12.5mm 5-Micron; Narrow-Bore 2.1×150mm 5μm). Subsequently, the mixture was loaded with a flow rate of 300μl/min for 1 h using a linear binary gradient of 0-80% buffer B (350 mmol/L KCl in solvent A, pH = 2.8). Eventually, a total of 6 SCX fractions were collected per iTRAQ set; and the absorbance at 210 and 280 nm was monitored.

### LC-MS/MS analysis

The mixed peptides, which were desalted with a PepMap C18 cartridge, were further separated by LC-20AD nano-RPLC (AB SCIEX) on the secondary RP analytical column (75μm x 15cm C18- 3μm 120 Å, ChromXP Eksigent). Peptides were subsequently eluted using the gradient conditions with phase B at a flow rate of 300 μL/min (5% phase B for 8 min, 25% phase B for 38 min, 40% phase B for 50 min, 90% phase B for 60 min, 2% phase B for 65 min, and 0 phase B for 70 min). Phase B was composed of 95% ACN and 0.1% formic acid.

Electrospray voltage of 2.5kV versus the inlet of the mass spectrometer (TripleTOF5600) was used. A hybrid quadrupole time-of-flight mass spectrometer (QStar hybrid LC/MS/MSQ-TOF, AB SCIEX, China) was operated in information dependent analysis mode for switching automatically between MS and MS/MS acquisition. MS spectra were acquired across the mass range of 400-1800 m/z in high-resolution mode using 250 msec accumulation time per spectrum. For information-dependent acquisition, survey scans were acquired in 250 msec—and as many as 35 product ion scans were collected if they exceeded a threshold of 150 counts per second (counts/s) with a 2+ to 5+ charge-state. Tandem mass spectral scanned from 100-2000 m/z in high-sensitivity mode with collision-induced dissociation. Dynamic exclusion was set for ½ of peak width (18s). The precursor was then refreshed off the exclusion list.

### Protein identification

The obtained LC-MS/ MS data were also analyzed by Sangon Biotech (Shanghai) Co., Ltd. Data were analyzed using the SEQUEST algorithm and comparing the results against the findings described in NCBI Human RefSeq database. The search parameters included iTRAQ labeling at N-terminus and lysine residues, cysteine modification by methyl methanethiosulfonate (MMTS), and digestion by trypsin. In our study, the cutoff value >1.3 and peptides >= 1 was applied towards protein identification. Proteins were considered as being differentially expressed if iTRAQ ratios were ≥2.0 or ≤0.5 in the carcinoma tissue group as compared to the pericarcinous tissue group. In addition, the *P* values <0.05 of iTRAQ ratios in at least one of the data sets was considered as being significantly differentially expressed.

### Bioinformatics analysis

The basic properties of the differentially expressed protein database were analyzed with GENENTOLOGY (http://www.pantherdb.org/), DAVID 6.7 (https://david.ncifcrf.gov/), and STRING (https://string-db.org/).

### Tissue microarray (TMA)

Tissue microarrays (TMAs) were constructed by the Department of Pathology, Basic Medical School of Lanzhou University. In addition, Shanghai Outdo Biotech Company (Shanghai, China) provided technical advice during this process. H&E-stained section of the TMA recipient block was prepared and reviewed to confirm the presence of intact neoplasm. The TMA contained a total of 122 point, of which 31 included both carcinoma foci and non-carcinoma foci. Every core was obtained from each paraffin-embedded CCA sample (using punch cores measuring 1.9 mm at their greatest diameter) from the non-necrotic area of carcinoma foci and/or non-carcinoma foci.

### Immunohistochemistry (IHC)

#### Antibodies

IHC was performed using the following antibodies, which were all obtained from Abcam ( Shanghai, China). Peroxiredoxin2 (PRDX2) was detected by Rabbit monoclonal Anti-Peroxiredoxin2 (ab109367). Biglycan (BGN) was detected by rabbit polyclonal Anti-Biglycan (ab49701). Lumican (LUM) was detected by rabbit monoclonal Anti-Lumican (ab168348). PPP3CA expression was examined by rabbit polyclonal Anti-Calcineurin A antibody (ab71149). SP-9001 SPlink Detection Kit (Biotin-Streptavidin HRP Detection Systems), 3,3' diaminobenzidine (DAB) Chromogen/HRP Substrate kit, and avidin-biotinylated horseradish peroxidase complex (ABC) were purchased from Zhongshan Golden Bridge Biotechnology Co. (Beijing, China).

#### Staining Procedure

IHC staining was performed on formalin fixed paraffin embedded tissue sections (3μm) and TMAs by an immunoperoxidase method using the avidin-biotinylated horseradish peroxidase (ABC) complex. Each section was deparaffinized with xylene and rehydrated in descending dilutions of ethanol and distilled water, respectively. Antigen retrieval was performed by the immersion of tissue sections and TMAs in pre-heated 10mM citrate buffer solution (pH 6.0) and maintaining heat at 120℃in a pressure cooker for 3 min. Endogenous peroxidase was blocked by incubating the sections in 0.3% hydrogen peroxidase at room temperature for 10 min. The sections and TMAs were washed in 0.01 mol/L phosphate-buffered saline (PBS, pH 7.4). 2% normal goat serum in PBS was applied for 10 min at room temperature to prevent non-specific staining.

In the staining using each antibody, the sections and TMAs were incubated with dilutions of the primary antibodies (PRDX2, 1:300; BGN, 1:100; LUM, 1:20; PPP3CA, 1:100) in PBS at 4℃ overnight. The sections were washed 3 times with PBS and incubated with the biotinylated secondary antibodies, and then were washed 3 times with PBS. All the sections then received ABC complex for 20 min at 37℃. After washing with PBS 3 times, the sections were finally reacted with DAB substrate for 10 min for visualization, rinsed with tap water, counterstained with hematoxylin, and mounted.

### Evaluation of the Results by Scoring

Immunoreactivity was evaluated independently by two pathologists who were blinded to the research design. Any discrepant scores were re-examined by both pathologists to reach a consensus score. Expression of PRDX2 in the cytoplasm was evaluated. In BGN, the cytoplasm and/or nucleus was evaluated. In LUM, membranous and cytoplasm was evaluated. For PPP3CA expression in cytoplasm was evaluated. A total immunostaining score (TIS) was calculated as the product of a percentage score and an intensity score as previously described 15. The percentage score described the estimated percentage of positively stained neoplastic cells (0: 0-5%; 1: 6-25%; 2: 26-50%; 3: 51-75%; 4: >75%). The intensity score represented the estimated staining intensity (0: no or marginal staining; 1: weak; 2: moderate; 3: strong). The TIS ranged from 0 to 12. We defined 0 as negative, 1-4 as weak positive, 5-8 as moderate positive, and 9-12 as strong positive.

### Statistical analysis

Statistical analysis was performed using the chi-square test, Fisher exact test, or *t*-test where appropriate. The overall survival (OS) of CCA patients was compared between the group with PPP3CA expression and the non-expressing group by using the Kaplan-Meier method; and differences between the survival curves were tested using the log-rank test. Univariate and multivariate survival analyses were performed using the Cox proportional hazards regression model. For the multivariate model, we used 0.10 as the cutoff *P*-value to select the analyzed factors from the univariate analysis data. A probability of a *P* less than 0.05 was considered to be statistically significant. All statistical analyses were conducted with the SPSS 17.0 software package (SPSS Inc., Chicago, IL).

## Results

### Proteins differentially expressed between primary CCA and pericarcinous tissue

**Figure 1:**
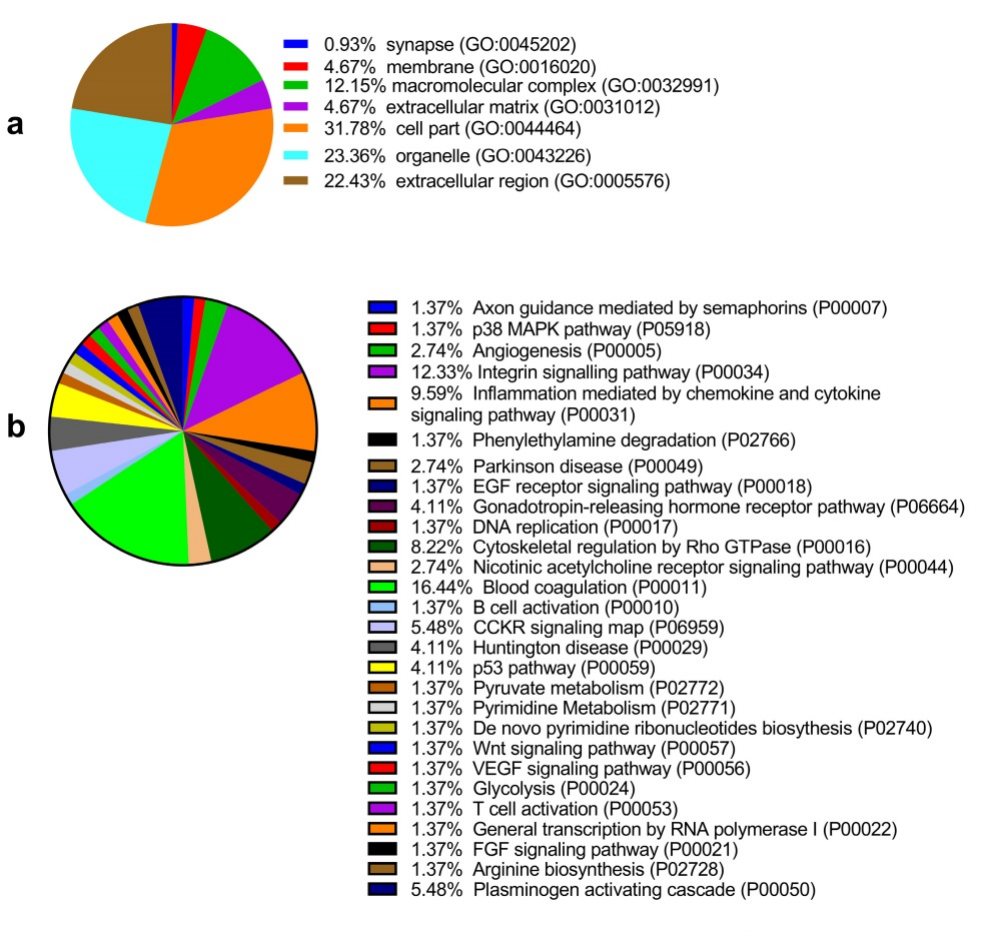

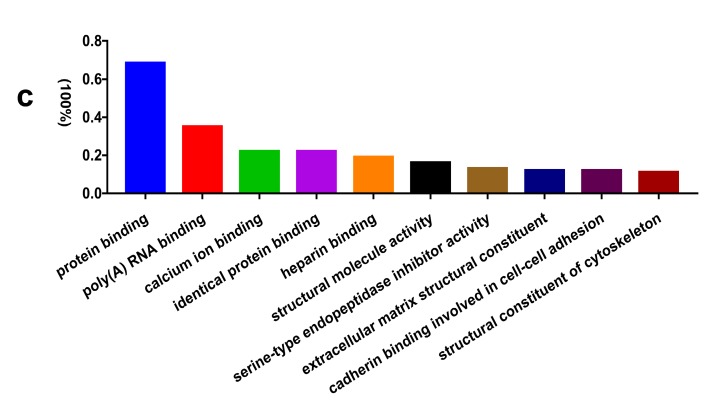

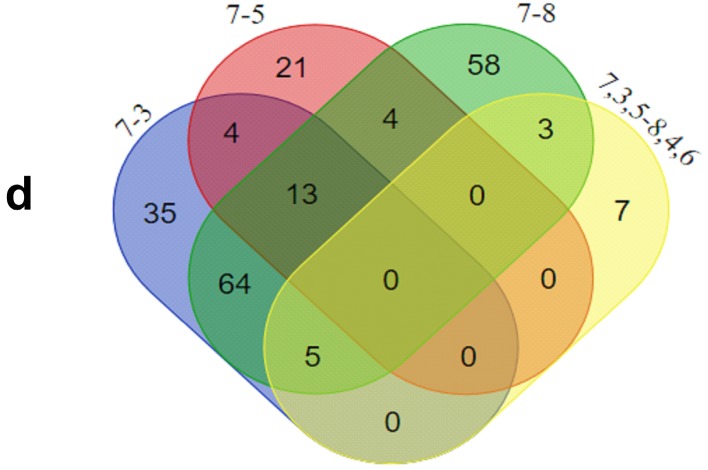

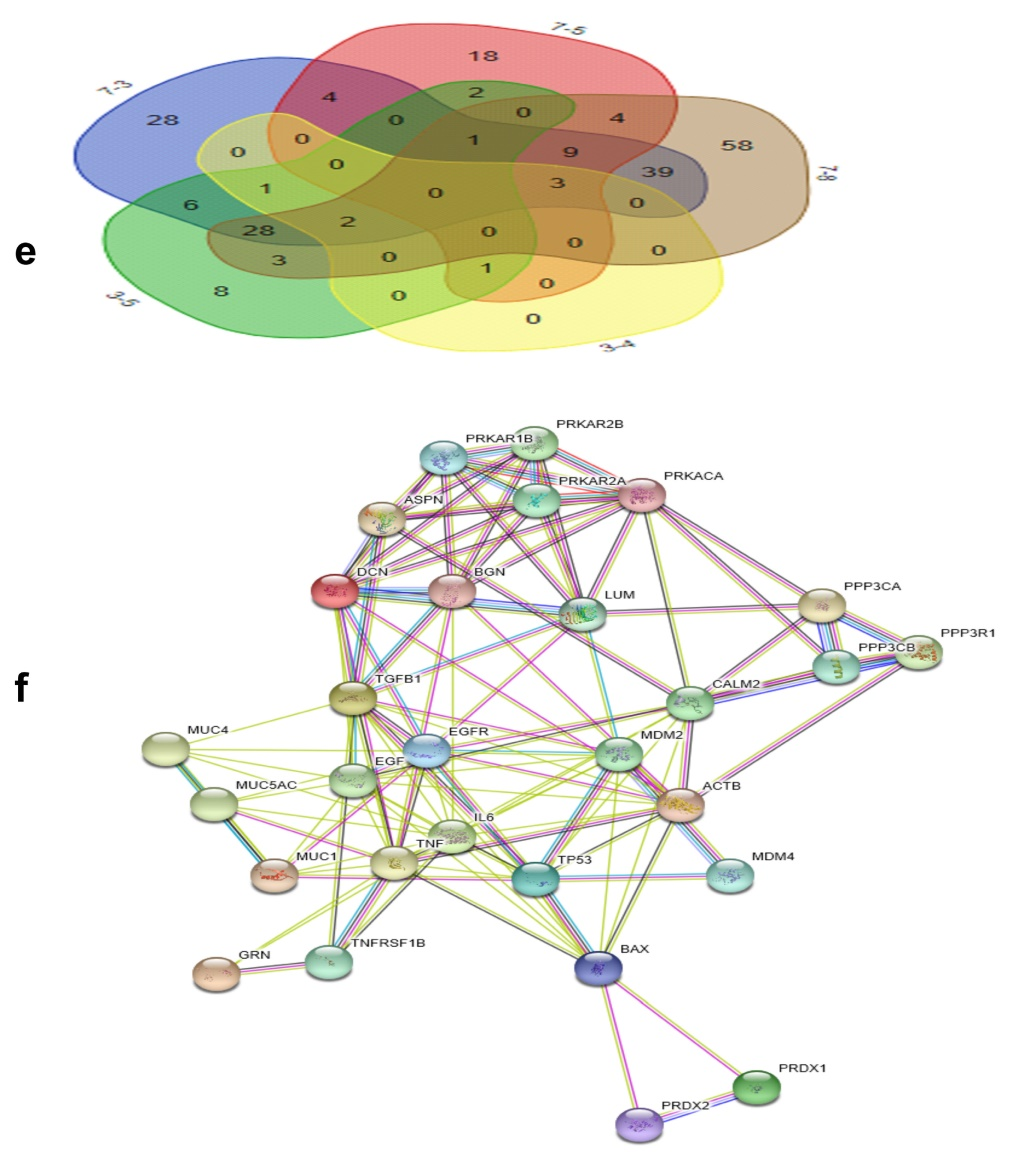
GO analysis of the differentially expressed proteins: The cellular component distribution of identified proteins (**a**); The pathways of these proteins involvement (**b**). The top ten biological processes enriched by DAVID (**c**). Venn diagram showing the intersections of identified proteins in different groups (**d, e**). The functional network of 19 differentially expressed proteins by searching in the STRING Database (**f**).

Among the 6 paired primary CCA and pericarcinous tissue, we identified 2,886 confidential proteins with a confidence interval (CI) ≥ 95% by using the iTRAQ technique. Among these, 233 proteins were differentially expressed in CCA tissue and pericarcinous tissue, which including 99 up-regulated and 134 down-regulated proteins. Supplementary Figure 1 shows the chromatographic separation of small molecules in CCA tissue. And a cluster heatmap (Supplementary Figure 2) was applied to visualize the gene expression data in CCA, which shows the up-regulated (red color) and down-regulated proteins (green color). Moreover, as **Fig. 1a** shows, these proteins can be classified into seven categories for cellular components using the PANTHER classification system. A majority of proteins identified were classified as being a cell part (31.78%), organelle (23.36%), from the extracellular region (22.43%), or cytoplasmic (26.90%) in nature. Other identified proteins were classified as originating in the macromolecular complex (12.15%), the extracellular matrix (4.67%), membranes (4.67%), and synapses (0.93%). GENEONTOLOGY analysis revealed that the pathways associated with these proteins can be classified into 28 groups. As shown in **Fig. 1b**, the top 5 five pathway groups were blood coagulation (16.44%), the integrin signaling pathway (12.33%), inflammation mediated by the chemokine and cytokine signaling pathway (9.59%), cytoskeletal regulation by Rho GTPase (8.22%), and either the CCKR signaling map (5.48%) or plasminogen activating cascade (5.48%).

In addition, functional annotation analysis showed that these proteins could be divided into 66 functional groups. The top ten functional groups consisted of the following: protein binding (68.3%), poly(A) RNA binding (35%), calcium ion binding (22%), identical protein binding (22%), heparin binding (19%), structural molecule activity (16%), serine-type endopeptidase inhibitor activity (13%), extracellular matrix structural constituent (12%), cadherin binding involved in cell-cell adhesion (12%), and structural constituent of cytoskeleton (11%) (**Fig. 1c**). Based on the functional process of differentially expressed proteins and Venn diagram results (**Fig. 1d, 1e**) which showed the intersections of differentially expressed proteins among different paired groups, we selected 18 significantly differentially expressed proteins. These are summarized in **Table 3.**

**Figure 2:**
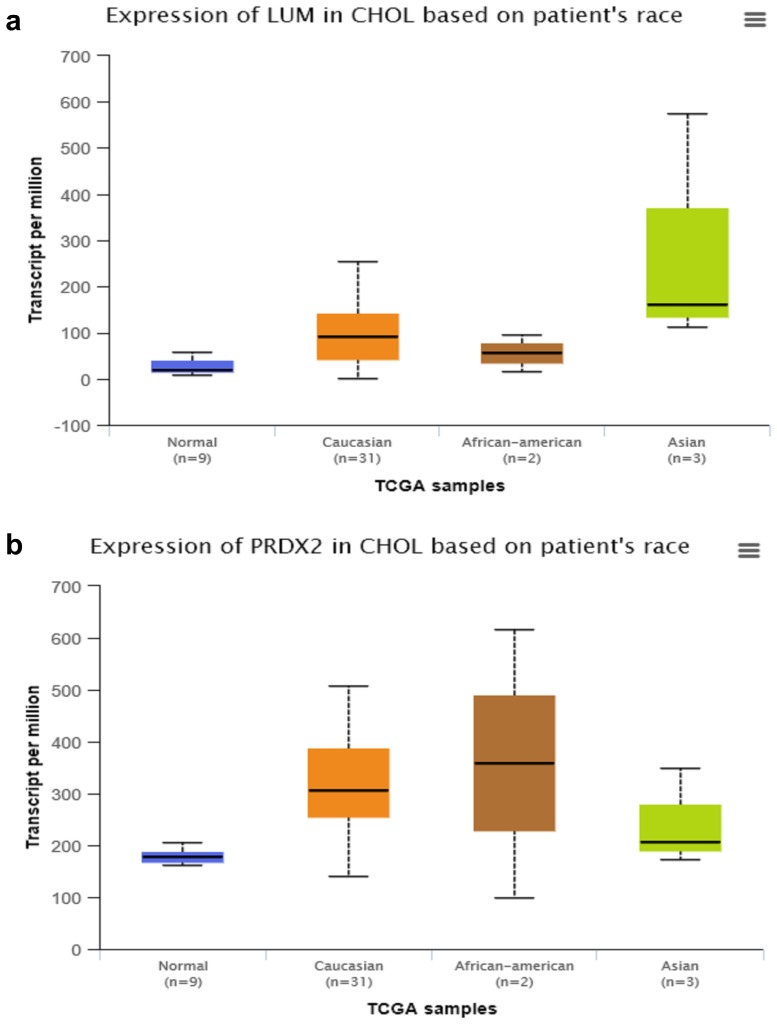

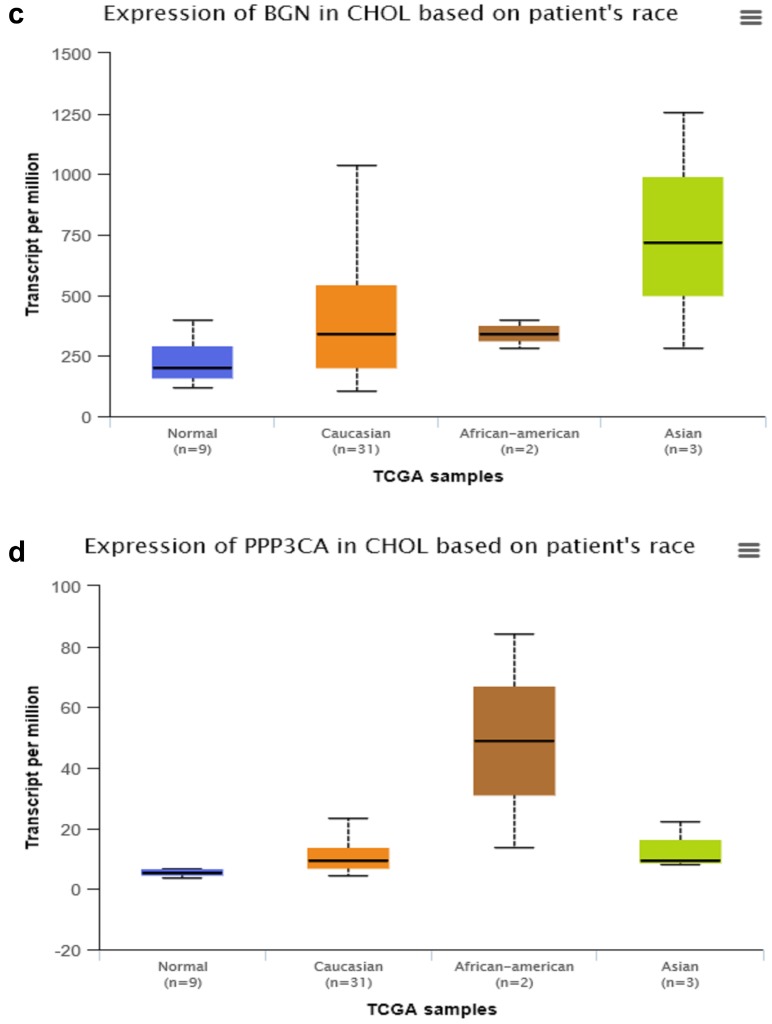
The comparison of expression of LUM (**a**), PRDX2 (**b**), BGN (**c**), and PPP3CA (**d**) in CCA based on the patient's race by searching the online TCGA database.

By searching STRING (https://string-db.org/), as **Fig. 1f** shows, we found the STRING interaction network of these 18 proteins. By means of literature review, we found that MUC1, MUC5AC, EGFR, TGFB1, and TNF, etc., have been demonstrated with prognostic and/or diagnostic value in relation to CCA 16 ,17. Finally, based on the functional process of the remaining proteins, we selected PRDX2, BGN, LUM, and PPP3CA as potential biomarkers for further examination of the in-tissue expression profile. In addition, by searching the online TCGA database (https://cancergenome.nih.gov/), we performed additional analyses of the expression of LUM, PRDX2, BGN, and PPP3CA in CCA based on the patient's race, which were depicted in **Fig. 2**, and the statistical significance for the comparison of the expression of LUM, PRDX2, BGN, and PPP3CA based on the patient's race is summarized in Supplementary table 1.

### Hematoxylin and eosin (H&E) staining

**Figure 3:**
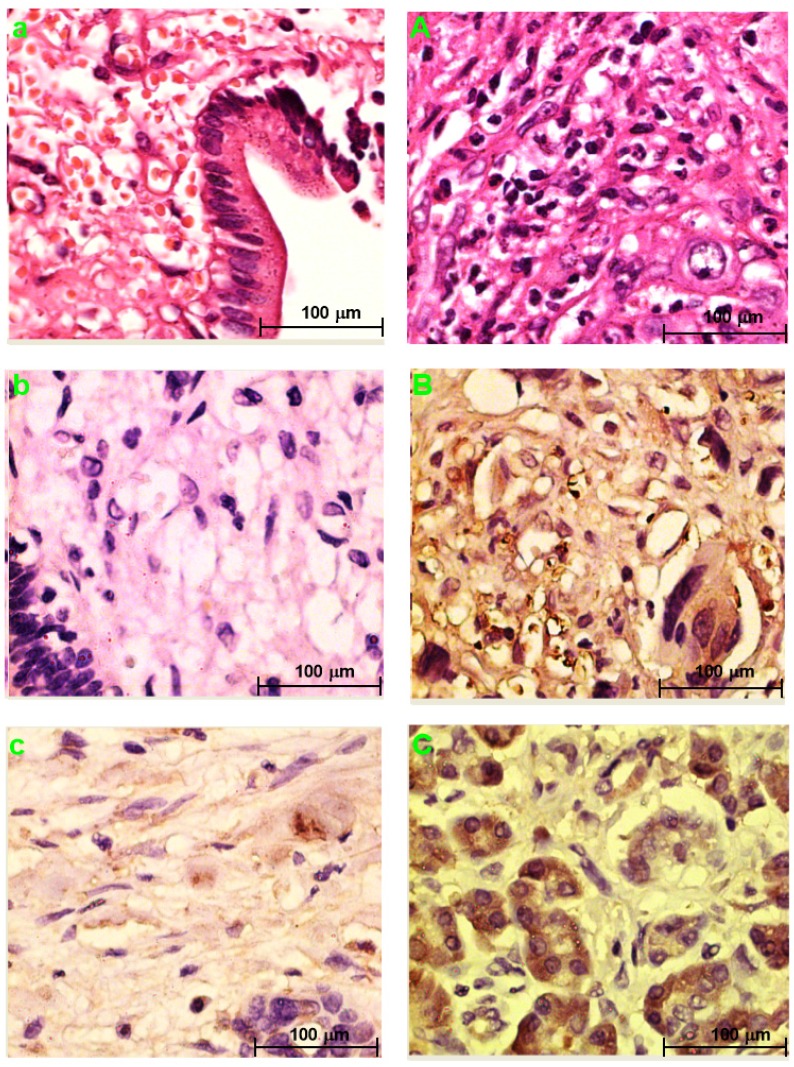

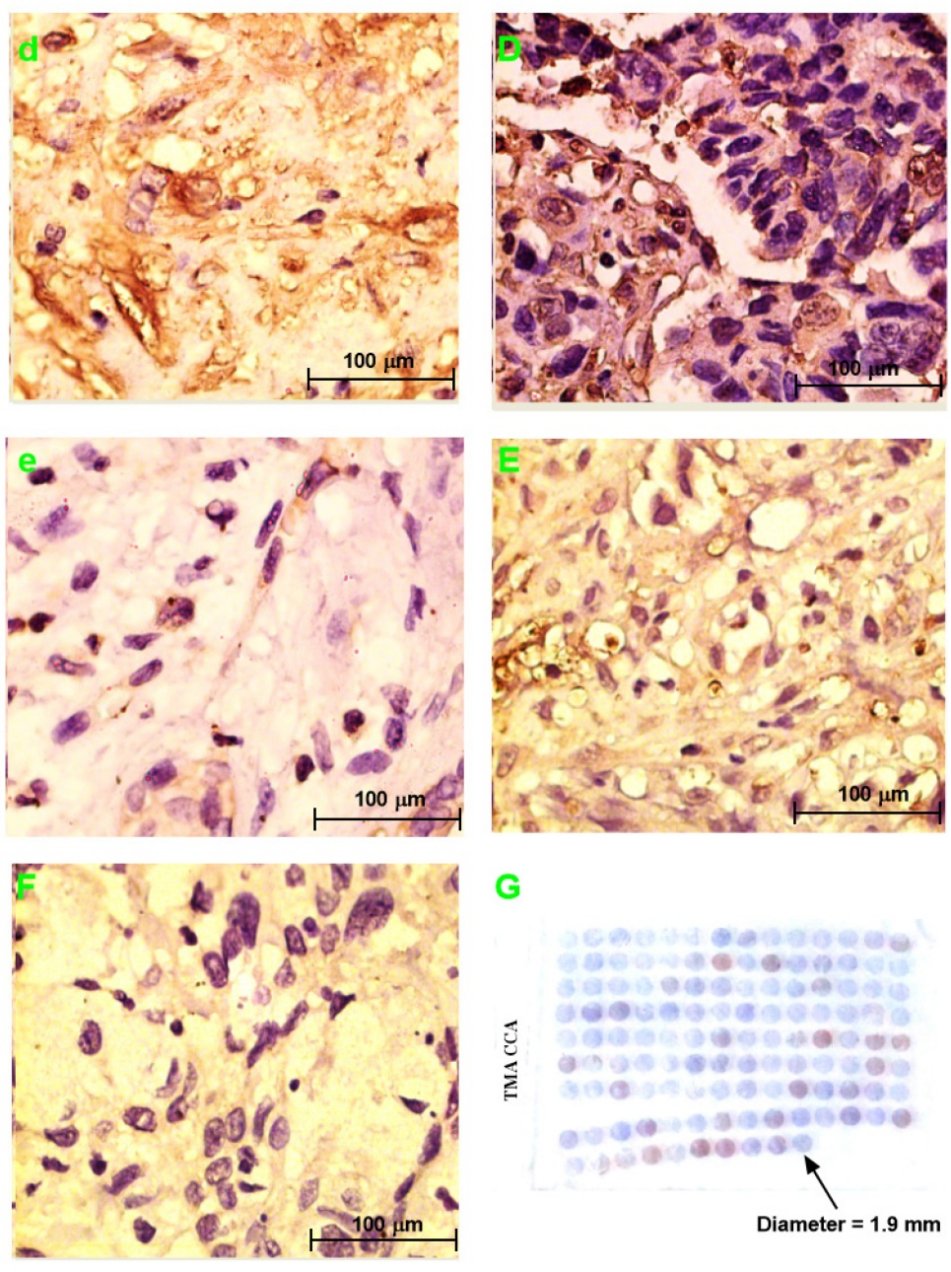
H&E staining section of choledochal cyst (**a**); H&E staining section of CCA (**A**). Original magnification ×200 (**a, A**). IHC staining section of the choledochal cyst: PRDX2 (**b**) and PPP3CA (**e**) are not expressed, whereas BGN lowly expressed in the cytoplasm (**c**), and LUM is expressed in the cell membrane and the cytoplasm (**d**). IHC staining section of the carcinoma: PRDX2 is expressed in the cytoplasm (**B**), while BGN is expressed in the cytoplasm and/or the nucleus (**C**). LUM is expressed in both the cell membrane and the cytoplasm (**D**). PPP3CA was highly expressed in cytoplasm (**E**) and lowly expressed in cytoplasm (**F**). Original magnification ×200 (**a-e**; **A-F**). The overall staining profile of PPP3CA expression in TMAs (**G**).

**Fig. 3a** and **Fig. 3A** showed the H&E-stained sections of choledochal cyst and CCA, respectively.

### PRDX2, LUM, BGN, or PPP3CA expression in choledochal cysts

PRDX2 was not expressed in choledochal cysts (**Fig. 3b**). BGN was lowly expressed in the cell cytoplasm in 1 (20%) of 5 of choledochal cysts tissues (**Fig. 3c**). LUM was expressed in the cell membrane and cytoplasm in 3 (60%) of 5 of choledochal cyst tissues (**Fig. 3d**); and PPP3CA was not expressed in choledochal cyst tissue (**Fig. 3e**).

### PRDX2, LUM, BGN, or PPP3CA expression in CCA

PRDX2 was in the cytoplasm of carcinoma cells in all 5 CCA cases (**Fig. 2B).** BGN was expressed in the cytoplasm and/or nucleus of carcinoma cells in 2 (40%) of 5 CCA cases (**Fig. 3C**). LUM was expressed in the cell membrane and cytoplasm of carcinoma cells 3(60%) of 5 CCA cases (**Fig. 3D**). PPP3CA was seen in the cytoplasm of carcinoma cells in 22(24.2%) of 91 CCA in TMA (**Fig. 3E, 3F**). The overall staining profile of PPP3CA expression in TMA is shown in **Fig. 3G**.

### The semi-quantitative analysis of the expression of PRDX2, BGN, LUM, and PPP3CA in CCA tissue and choledocal cyst tissue

**Figure 4:**
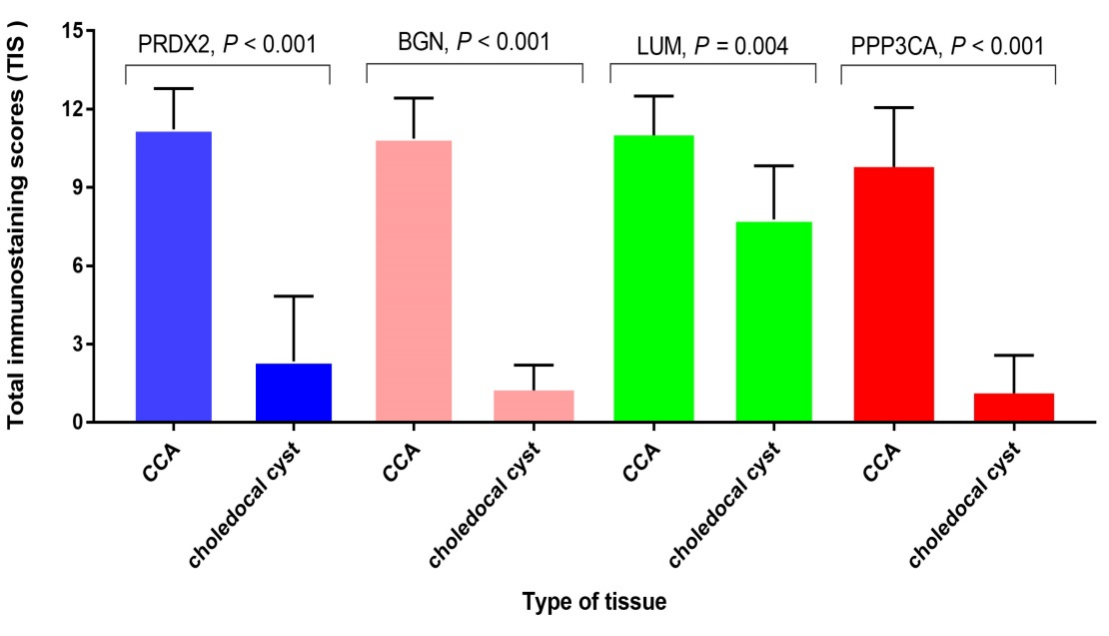
The semi-quantitative analysis of the expression of PRDX2, BGN, LUM, and PPP3CA in CCA tissue and choledocal csyt tissue.

As** Fig. 4** shows, the expression of PRDX2, BGN, and PPP3CA was significantly lower in choledocal cyst tissue than in CCA tissue (*P* < 0.001). Furthermore, in LUM, there was also a significant difference between CCA tissue and choledocal tissue (*P* = 0.004).

### Relationship between PPP3CA expression and clinicopathological features of CCA patients

In the analysis of the PPP3CA expression and of clinicopathological features, 3 patients were excluded since these patients were lost during follow-up. PPP3CA expression was not related to most of the clinicopathological features included in **Table 4**.

### Relationship between PPP3CA expression and overall survival rate

**Figure 5:**
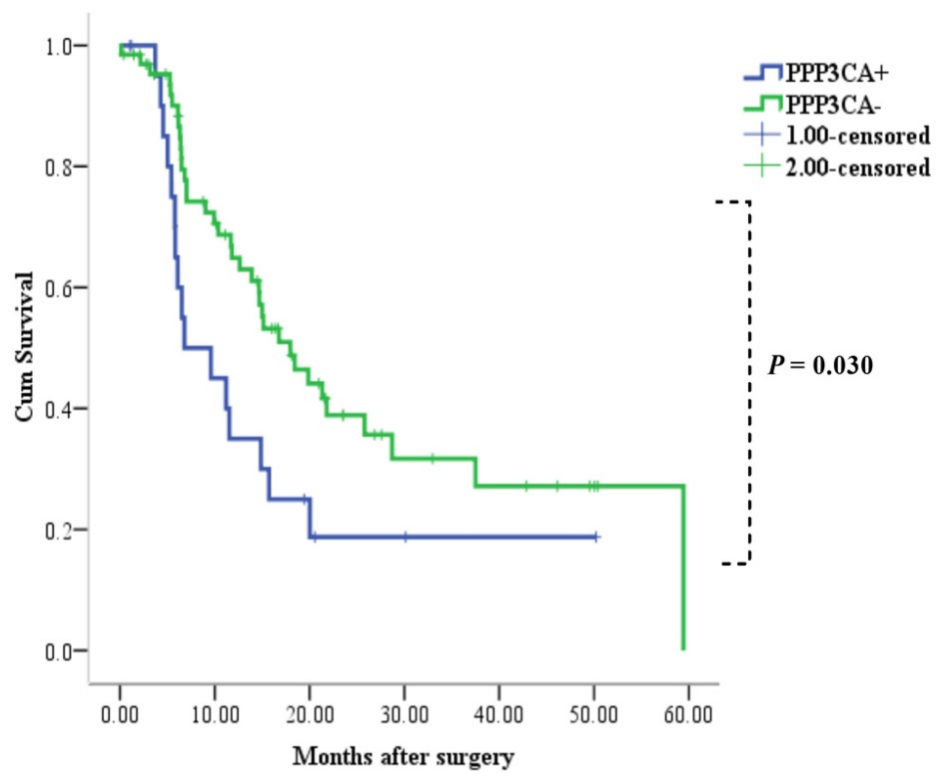
Correlation between PPP3CA expression and cumulative survival rate in patients with CCA (Kaplan-Meier method). The survival rate of patients with PPP3CA positive was poorer than that of patients with PPP3CA negative.

Among the 88 patients examined, 52 patients died during the follow-up period. Median and mean length of survival for CCA who underwent surgery therapy was 14.9 and 24.4 months, respectively. The 1- and 3-year OS rates for patients with positive PPP3CA expression were 33% and 19%, respectively. The 1- and 3-year OS rates for patients with PPP3CA negative expression were 65% and 32%, respectively. The survival rate of patients with PPP3CA positive expression (n=22) was significantly poorer than for those with PPP3CA negative expression (n=66) (*P*= 0.030, log-rank test; **Fig. 5**).

### Univariate and multivariate analysis of prognostic factors

The univariate analysis of prognostic factors of CCA is summarized in** Table 5a**. Tumor size (*P* = 0.002), vascular invasion (*P* = 0.001), histological grading (*P* = 0.011), and PPP3CA expression (*P* = 0.033) were statistically significant risk factors affecting the outcomes of CCA patients. In multivariate analysis, as summarized in **Table 5b**, vascular invasion (*P* = 0.012) and PPP3CA expression (*P* = 0.009) were found to be statistically significant independent risk factors for the OS of CCA patients.

## Discussion

Surgical resection with histologically negative resection margins and/or liver transplantation was the only effective treatment option for CCA patients with a long survival rate, whereas chemotherapies were widely reported to remain poorly characterized and virtually palliative given the marked chemoresistance of this heterogeneity group of cancer 17,18. Currently, there are few biomarkers with high diagnostic and/or prognostic capability for detecting CCAs or for predicting the prognosis of CCA patients.

In our study, we used the iTRAQ proteomic technique to find the differentially expressed proteins between the matched CCA and pericarcinous tissue. 233 differentially expressed proteins were identified in the initial search. Compared with pericarcinous tissues, 99 proteins were up-regulated and 134 proteins were down-regulated in CCA cases. Most of these proteins were involved in blood coagulation (16.44%), the integrin signaling pathway (12.33%), inflammation mediated by the chemokine and cytokine signaling pathway (9.59%), cytoskeletal regulation by Rho GTPase (8.22%), and the CCKR signaling map (5.48%), etc. Based on the results of the bioinformatic analysis and the literature review, we selected PRDX2, BGN, LUM, and PPP3CA as novel candidate biomarkers of CCA, as the expression profiles of these proteins in CCA were rarely reported. We further examined the expression profile of the selected biomarker in CCA tissue samples by using IHC. In addition, we assessed the PPP3CA expression rate in a total of 91 CCA cases and investigated the prognostic role of PPP3CA in CCA patients.

PRDX-2, a typical 2-Cys thioredoxin peroxidase and a cellular antioxidant, which have been considered as being a tumor-suppressing protein with the feature of regulating levels of hydrogen peroxide and mediating several signal transduction pathways which are linked to the regulation of cell proliferation, differentiation, and apoptosis 19-21. In our study, we firstly found that the expression pattern of PRDX-2 in CCA was down-regulated as compared with that in pericarcineous samples using iTRAQ-based quantitative proteomic analysis. In addition, as with the increased expression of PRDX2 in colorectal cancers, B cell‑derived primary lymphoma cells, and cervical cancer, etc. 22, similarly, we firstly found that PRDX-2 was positively expressed CCA cases but not expressed in choledochal cyst tissues, that is, the expression profile of PRDX2 in CCA is increased as compared with its expression in choledochal cyst tissues. However, the expression profile of PRDX2 in carcinomas is not always increased, given that a decreased expression of PRDX2 in melanomas has been demonstrated 23. PRDX2 can show a tumor‑promoting effect or tumor‑suppressive function based on tumor type. Further study needs to be performed to elucidate the mechanism of PRDX2 in the development CCA.

BGN, a classical type of extracellular matrix protein, is normally secreted from extracellular matrix fibroblasts, and facilitates the assembly of collagen fibrils and the bone matrix 24. The increased expression of BGN induces the activation of the NF-ҡB pathway via TLR2/4 signaling. The activation of NF-ҡB is associated with the aggressive growth of tumors and contributes to the resistance to chemotherapies during cancer treatments 25 ,26. Moreover, BGN up-regulation has been demonstrated in many malignancies such as pancreatic adenocarcinoma 27, prostate cancer 28, and colon cancers 24, etc. In addition, Nishino et al. 29 previously reported that BGN has the capacity of differentiating intrahepatic CCA from hepatocarcinoma or metastatic adenocarcinoma. In our study, we firstly found that the expression profile of BGN in extrahepatic cholangiocarcinoma was increased as compared with its expression in choledocal cysts. CCA classified as intrahepatic CCA, perihilar CCA, and distal CCA based on its anatomical location—which is associated with significant inter-tumor and intra-tumor differences that can affect pathogenesis and therapeutic outcome. The function of BGN in the development of CCA is still unclear. Future studies need to enucleate the molecular function of BGN in the development CCA, particularly based on the classification of CCA.

LUM, a Class II mall leucine-rich proteoglycans, is a key regulator of the organization of tumor matrix and cancer cell-matrix interactions due to its effects on collagen fibrillogenesis and degradation, which binds to cell membrane integrins and receptors and eventually result in the modulation of downstream signaling pathways 30. The expression of LUM in colorectal cancer 31 and pancreatic cancer 32 has been correlated with poor outcomes in both, Recently, Chen et al. reported that LUM could serve as an independent prognosticative factor in the prediction of gastric cancer 33. Currently no published reports have focused on investigating the correlation between LUM and CCA. In the present study, iTRAQ-based quantitative proteomic analysis suggested that the expression pattern of LUM in CCA is down-regulated as compared with its expression in pericarcinous tissue; we further found that the expression profile of LUM in CCA tissue was higher than its expression in choledocal cyst tissue (**Fig. 4**). The function of the increased expression of LUM in the prognosis of CCA, is unclear since the role of LUM in carcinomas can show differences based on the type of tumor involved.

PPP3CA, also known as calcineurin A, a catalytic subunit of calcineurin, contains one catalytic domain and three regulatory domains (including calcineurin B binding, calmodulin binding, and auto-inhibitory domains) 34. It is encoded by the PPP3CA gene located on chromosome 4q24, and it is responsible for the interaction of calcineurin with Ca^2+^/ calmodulin 35 ,36. Overexpression of the calcineurin family of secretory integrin-binding proteins has been demonstrated to have a correlation with tumor cell adhesion, proliferation, invasion and migration 37 ,38. Furthermore, PPP3CA expression has been reported to be elevated in breast cancer 39, lung cancer 40, and prostate cancer 41.

In our study, we firstly found that the expression of PPP3CA in CCA tissue was up-regulated as compared with its expression in paired pericarcinous tissue by using the iTRAQ proteomic technique. Further, using the Kaplan-Meier analysis (**Fig. 5**), we found that PPP3CA expression was significantly related to poor survival of the CCA patients, and that the PPP3CA expression is a statistically significant, independent, poor prognostic factor for CCA patients. That is, the outcomes after surgery were significantly poorer in the patients with PPP3CA-positive expression than in those with PPP3CA-negative expression. Tumor-negative resection margins (histologically); the absence of vascular invasion and the presence of lymph node metastasis; MUC1-negative expression, etc., have been reported as being prognostic factors in patients with resectable CCA 4 ,11 ,17. In our study, the univariate analysis for prognostic factors showed that tumor size, vascular invasion, histological grading, and PPP3CA expression were significant prognostic factors; whereas, in the multivariate analysis, PPP3CA expression, as well as vascular invasion, were independent risk factors for poor prognosis of this intractable disease.

PPP3CA, a protein-encoding gene, has been correlated with tumor development such as pancreatic carcinoma and breast cancer by partaking in the Wnt signaling pathway 39 ,42. In addition, in breast cancers, over-expression of PPP3CA in MCF-7 human breast cancer cells has been shown to up-regulate MAPK and NFkB signaling—promoting cell survival and chemoresistance 43, as well as promoting cell growth, migration, and angiogenesis in vivo 44. Tumor-associated macrophages activate the canonical Wnt-β-catenin pathway in tumor cells and thereby participate in CCA development by means of the production of Wnt ligands (Wnt3a and Wnt7b) 45. The depletion of tumor-associated macrophages or the inhibition of Wnt signaling with Wnt inhibitors both in vitro and in mouse and rat CCA models significantly reduced CCA proliferation and increased apoptosis, therby resulting in tumour regression 46. Furthermore, the MAPK pathway - activated by IL-6, which is constitutively secreted by CCA cells - has been correlated with cholangiocyte growth 47. The exact regulatory mechanisms of PPP3CA in CCA is unclear but may be due to 'parking' in the Wnt signaling pathway and/or MAPK pathway.

In conclusion, we found that PPP3CA, PRDX2, BGN, and LUM were differentially expressed in CCA tissue as compared with their expression in pericarcinous tissue. Expression of PPP3CA in CCA is an independent poor prognostic factor and is a useful marker for predicting the outcomes of CCA patients with surgical resection.

Due to the limitation of funding sources, we only assessed the prognostic role of PPP3CA in CCA patients. Further studies need to verify these findings and investigate the exact molecular pathways and interaction mechanisms of those proteins identified as playing roles in the development of CCA.

## Supplementary Material

Supplementary figures and tables.

## Figures and Tables

**Table 1:** Summary of The Clinicopathological Features of Individual Patients Collected for iTRAQ

Code	Pathology diagnosis	Histopathology	Lymph node metastasis	Gross appearance	Age (yrs)	Gender
1	Extrahepatic CCA	Moderately differentiated adenocarcinoma	Positive	Mass-forming	58	Male
2	Extrahepatic CCA	Moderately differentiated adenocarcinoma	Negative	periductal infiltrating	72	Male
3	Extrahepatic CCA	Moderately differentiated adenocarcinoma	Negative	periductal infiltrating	75	Male
4	Extrahepatic CCA	Poorly differentiated adenocarcinoma	Positive	periductal infiltrating	51	Male
5	Extrahepatic CCA	Well differentiated adenocarcinoma	Positive	intraductal growing	51	Female
6	Extrahepatic CCA	Poorly differentiated adenocarcinoma	Negative	Mass-forming	55	Female

CCA, cholangiocarcinoma

**Table 2:** Clinicopathological Features of CCA Patients

Age, mean (year, range)	51.2 (32-76)
**Gender (M : F), n (%)**	48 (52.7) : 43(47.3)
**T-stage, (n)%**	
T1	7 (7.7)
T2	62 (68.1)
T3	19 (20.9)
T4	3 (3.3)
**Histological grade, n(%)**	
Poorly	12 (13.2)
Moderate	70 (76.9)
Well	9 (9.9)
**Lymph node metastasis, n(%)**	
N0	60 (65.9)
N1	31 (34.1)
**Vascular invasion, n(%)**	
Negative	61 (67.0)
Positive	30 (33.0)
**Tumor size (cm), n(%)**	
>3.5	32 (35.2)
≤3.5	59 (64.8)
**Median follow up time (months, range)**	11.17 (0.13-59.43)

**Table 3:** The Basic Properties of Selected Protein Screened from Proteomics

Accession No.	Gene symbol	Sequence coverage (%)	Peptides	Gene ID
sp|P51884|LUM_HUMAN	LUM	57.1	62	4060
sp|Q07812|BAX_HUMAN	BAX	32.3	3	581
sp|P98088|MUC5A_HUMAN	MUC5AC	2.7	2	4586
tr|A0A0C4DGW3_HUMAN	MUC1	8.2	3	4582
sp|P21810|PGS1_HUMAN	BGN	63.3	64	633
sp|P07585|PGS2_HUMAN	DCN	28	69	1634
sp|Q9BXN1|ASPN_HUMAN	ASPN	59	36	54829
sp|P17612|KAPCA_HUMAN	PRKACA	13.1	3	5566
sp|Q08209|PP2BA_HUMAN	PPP3CA	20.4	8	5530
sp|P00533|EGFR_HUMAN	EGFR	3.5	2	1956
sp|P32119|PRDX2_HUMAN	PRDX2	75.3	47	7001
sp|Q06830|PRDX1_HUMAN	PRDX1	62.3	24	5052
sp|O43294|TGFI1_HUMAN	TGFB1	18.9	8	7040
sp|P28799|GRN_HUMAN	GRN	15	8	2896
sp|Q03169|TNAP2_HUMAN	TNF	18.2	5	7124
sp|P60709|ACTB_HUMAN	ACTB	88.5	490	60
tr|H0Y7A7|H0Y7A7_HUMAN	CALM2	57.2	28	805
sp|P16298|PP2BB_HUMAN	PPP3CB	20.6	6	5532

**Table 4:** Summary of The Data on The Expression of PPP3CA in Clinicopathological Features of 88 Cases of CCA

Category	No. Patients (%)	PPP3CA(%)		P value
		Positive (≥ 1+)	Negative (0)	
**Gender**				
Male	45(51.1)	11(24.4)	34(75.6)	0.902
Female	43(48.9)	11(25.6)	32(76.4)	
**Age** (**yrs**)				
>55	50(56.8)	14(63.6)	36(54.5)	0.456
≤55	38(43.2)	8(36.4)	30(45.5)	
T-**stage** (**AJCC7th**)				
T1,2	67(76.1)	17(77.3)	50(75.8)	0.885
T3,4	21(23.9)	5(22.7)	16(24.2)	
**Lymph node metastasis**				
N0	58(65.9)	11(50.0)	47(71.2)	0.069
N1	30(34.1)	11(50.0)	19(28.8)	
**Vascular invasion**				
Negative	29(33.0)	15(68.2)	44(66.7)	0.896
Positive	59(67.0)	7(31.8)	22(33.3)	
**Tumor size (cm)**				
>3.5	31(35.2)	11(50.0)	20(30.3)	0.094
≤3.5	57(64.8)	11(50.0)	46(69.7)	
**Histological grading**				
Poorly	12(13.6)	3(13.6)	9(13.6)	0.269
Moderate	68(77.3)	15(68.2)	53(80.3)	
Well	8(9.1)	4(18.2)	4(6.1)	
**Survival (mo.)**				
>12	41(46.6)	7(31.8)	34(51.5)	0.109
≤12	47(53.4)	15(68.2)	32(48.5)	

**Table 5a:** Univariate Analysis of Prognostic Factors

Variable	Hazard ratio	95%confidence interval	P value
**Gender**			
Male (n = 45)	1		
Female (n = 43)	0.801	0.460-1.398	0.435
**Age (yrs)**			
≤55 (n = 38)	1		
>55 (n = 50)	1.007	0.566-1.791	0.981
**T-stage (AJCC7th)**			
T1,2 (n = 67)	1		
T3,4 (n = 21)	1.547	0.862-2.776	0.143
**Lymph node metastasis**			
N1 (n = 30)	1		
N0 (n = 58)	0.604	0.343-1.064	0.081
**Vascular invasion**			
Negative (n = 29)	1		
Positive (n = 59)	2.631	1.511-4.580	0.001**
**Tumor size (cm)**			
≤3.5 (n = 57)	1		
>3.5 (n = 31)	2.435	1.384-4.284	0.002**
**Histological grading**			
Poorly (12)	1		
Moderate (68)	0.162	0.040-0.655	0.011*
Well (8)	0.473	0.199-1.120	0.089
**PPP3CA**			
Negative (66)	1		
Positive(22)	1.906	1.052-3.454	0.033*

* P < 0.05; ** P <0.01

**Table 5b:** Multivariate Analysis of Prognostic Factors

Variable	Hazard ratio	95%confidence interval	P value
**Vascular invasion**			
Negative (n = 29)	1		
Positive (n = 59)	2.204	1.191-4.079	0.012*
**Tumor size (cm)**			
≤3.5 (n = 57)	1		
>3.5 (n = 31)	1.694	0.914-3.139	0.094
**Histological grading**			
Well/Moderate	1		
Poorly	2.664	0.806-8.808	0.108
**PPP3CA**			
Negative	1		
positive	2.279	1.228-4.229	0.009**

* *P* < 0.05; ** *P* <0.01

## Abbreviations

CCA: Cholangiocarcinoma
iTRAQ: Isobaric tags for relative and absolute quantitation
PRDX2: Peroxiredoxin2
BGN: Biglycan
LUM: Lumican
PPP3CA: Serine/threonine-protein phosphatase 2B catalytic subunit alpha isoform
IHC: Immunohistochemistry
OS: Overall survival
LC-MS: Liquid chromatography-mass spectrometry
CA19-9: Carbohydrate antigen 19-9
TMA: Tissue microarray
SCX: Strong cation exchange
H&E: Hematoxylin and eosin
DAB: Diaminobenzidine
ABC: Avidin-biotinylated horseradish peroxidase complex
