# Supplementary material for: Quantitative Proteomic Analysis and Evaluation of the Potential Prognostic Biomarkers in Cholangiocarcinoma
Source: J Cancer. 2019 Jul 5;10(17):3985–99. doi: 10.7150/jca.29354 (PMC6692627; doi:10.7150/jca.29354)
Supplement: Supplementary file 1 — Supplementary figures and tables. [file jcav10p3985s1.pdf]

Supplementary Figure legends:

Supplementary Figure 1. A diagram of chromatographic separation of small molecules in cholangicarcinoma.

Supplementary Figure 2a, 2b. Cluster heatmaps visualizing the gene expression data in cholangiocarcinoma. (Rows represent the the expression level of proteins in each group, columns represent individual protein

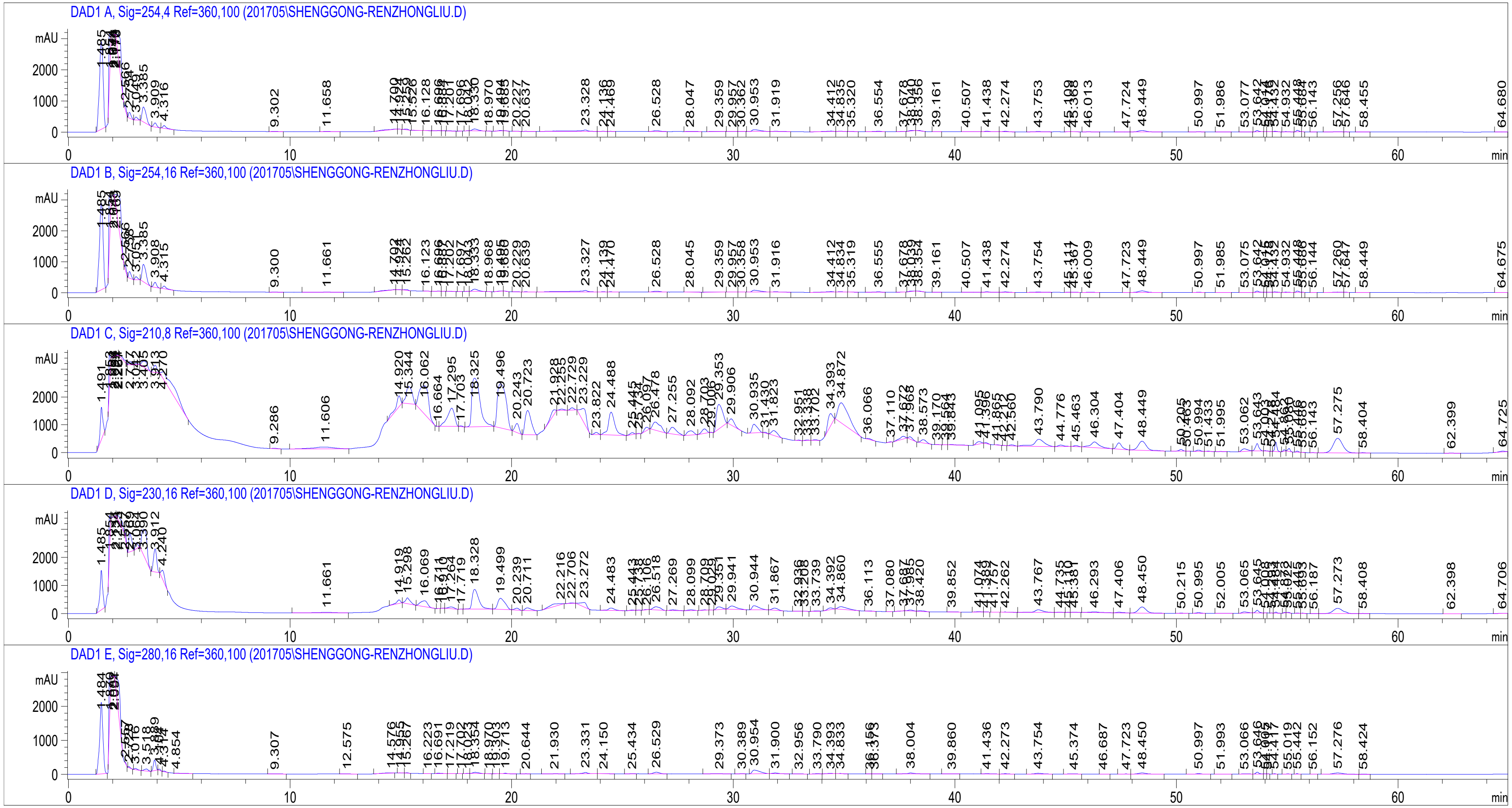

Supplementary Figure 1

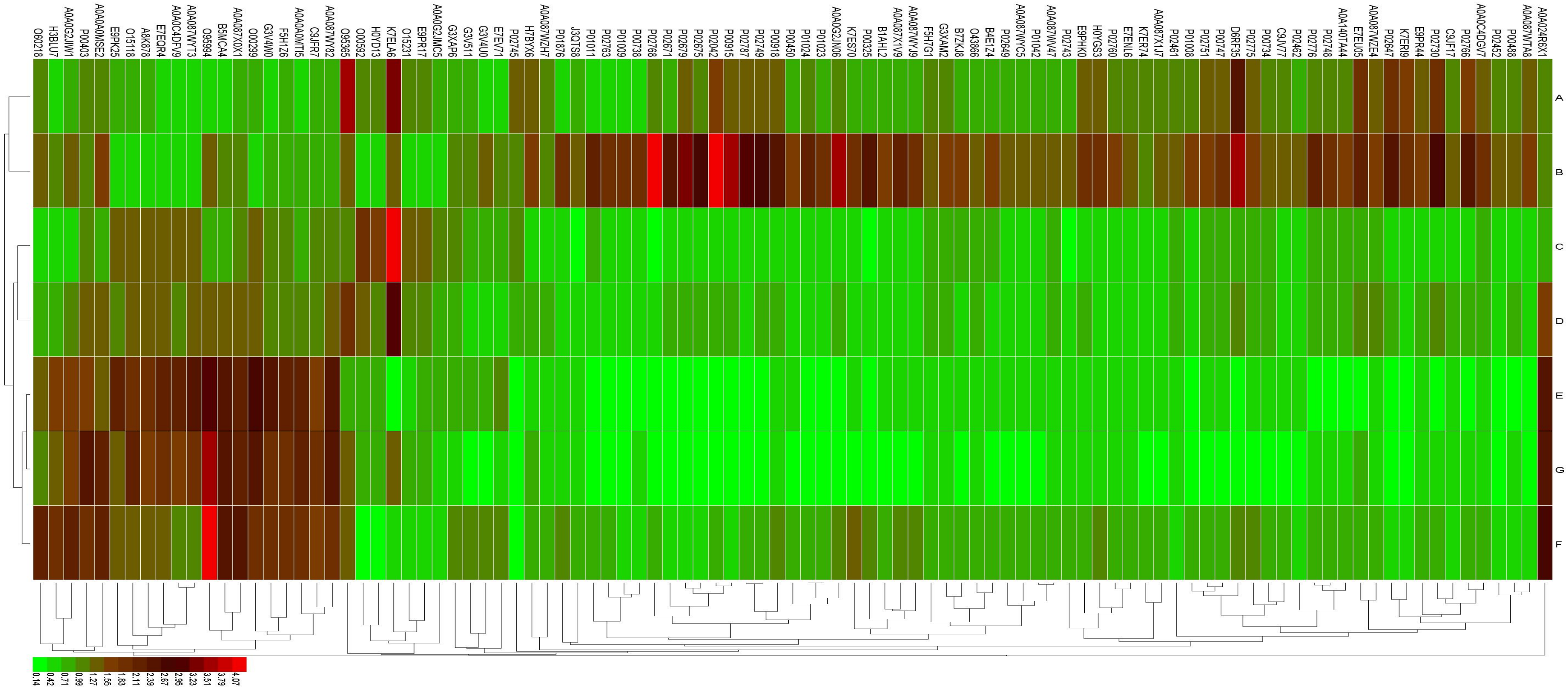

Supplementary Figure 2a

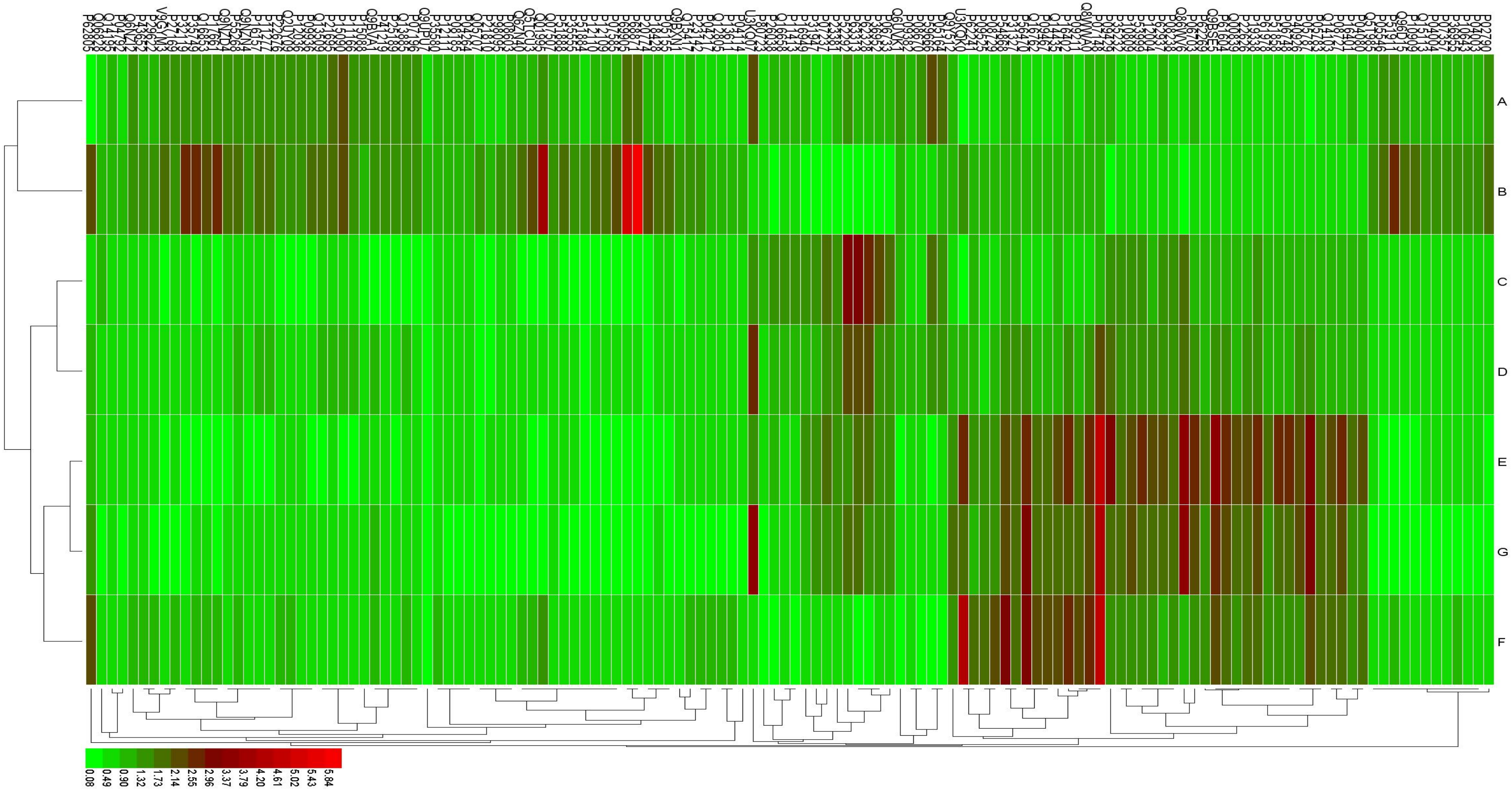

Supplementary Figure 2b
